# Gravel and Stone. Clinical Remarks on Their Formation and Treatment

**Published:** 1907-12-07

**Authors:** Reginald Harrison

**Affiliations:** Past Vice-President and Hunterian Professor, Royal College of Surgeons.


### GRAVEL AND STONE.

### Clinical Remarks on their Formation and Treatment.

By REGINALD HARRISON, F.R.C.S., Past Vice-President and Hunterian Professor, Royal College of Surgeons.

In late years important advances have been made in the treatment of calculous disease of the urinary organs. The point I propose to expand and give prominence to is, the more general recognition and practical application as a working basis for the purposes of prevention and treatment of Rainey's views relative to the formation of gravel and stone.

It seems remarkable that, in the various discussions which have taken place within my recollection as to the causation and formation of urinary calculi, little or no reference has been made to Rainey's original and important work on this subject. It is, however, a matter of congratulation that these investigations, and those of Ord and Vandyke Carter, are gradually finding their way into modern text-books, as, for example, the recent works of Keyes and Fowler in America.

In 1851, Rainey, of St. Thomas's Hospital,* demonstrated a mode of making calculi artificially by what he then, and subsequently, described in a published treatise as molecular coalescence, "the general outcome of which," to use Mr. Plowright's † words, "was to demonstrate the fact that the presence of a colloid at the moment various bodies pass into their crystalline forms so modifies the cystalloid that instead of becoming a crystal the body separates in a coherent form."

It has often struck me that the repetition of Rainey's interesting experiments in the laboratory or classroom by students engaged in pathological research would prove the means of directing attention to a subject upon which more information should be available. I have on several occasions, when conducting pass examinations in surgery for diplomas and degrees, tested candidates on this matter, but without receiving any very satisfactory replies.

The process of molecular coalescence not only explains the formation of shells and calculi, but

* "Precise Directions for making Artificial Calculi by Molecular Coalescence." Trans. Royal Microscopical Soc. Vol. vi. and Churchill, London. 1858.

† Medical Times and Gazette. Oct. 10, 1885.

also—1. Throws light on the comparative incidence of urinary stone relative to the population; 2. Offers a rational principle upon which preventive treatment may be applied to these disorders; and, 3. Supplies an intelligible reason for the adoption of methods of practice which hitherto have been largely empirical.

1. It is rather startling to realise what might happen if the concretion of certain constituents of normal urine were a simple process which could be readily brought about.

A healthy adult excretes in 48 days more than sufficient uric acid to form a stone in his bladder not less than half an ounce in weight, so that there must be contributing factors in calculus formation, beside the constituent ingredient. If mere excesses of certain substances, such as urates and uric acid, could alone act as causes of stone, these affections would doubtless be far more common than they are.

That the process of stone making is a complex one is obvious to anyone who repeats Rainey's experiments. These tend to show that the preponderance of stone cases in certain districts is due not alone to excess of the constituent elements of stones —for instance, uric acid or oxalate of lime—but also to a greater liability in the district concerned to the concurrence of all the conditions or factors necessary for the process of molecular coalescence. This is a distinction with an important difference. In places and districts where stone cases at one time were very prevalent and have ceased to be so it has not been shown that a change for the better has been brought about by any corresponding diminution in the amount of uric acid secreted, but rather to a general improvement in the sanitary surroundings of the inhabitants.

2. I pass on to notice, in the second place, how this consideration may be applied artificially to individual cases by maintaining an equal diffusion of the urinary colloids. Mr. Plowright has shown the influence that common salt exercises in the prevention of gravel and stone, and illustrates his argument by the well-known rarity of the latter amongst sailors and residents in salt-producing districts. The explanation, as experimentally determined by Dr. Ord, is "that whatever tends to keep the colloids diffused will oppose the formation of calculus." It seems not unreasonable to suppose that there may be other ways of effecting a similar interference with the conditions necessary for molecular coalescence. In an article in the *Lancet* (February 9, 1901) I referred to some observations I had been making on certain drugs which had been employed somewhat extensively in connection with the prevention of calculous disorders—namely, magnesian borocitrate and a preparation known then and now as "Dutch drops." Both of these preparations were at one time held in high repute in connection with the treatment of gravel and stone, and have undeservedly, as I believe, dropped out of use, as many other excellent remedies have done.

My attention was first called to the borocitrate of magnesia (pulv. magnes. boro-citratis co.: dose, ʒj. in water two or three times a day—Martindale's Extra Pharmacopœia) by a paper of Dr. Koehler, of Kosten.‡ It is prepared by dissolving a natural borate of magnesia found at Stassfurt and elsewhere in citric acid. From various papers which preceded that of Dr. Koehler boracite appears to have formed the basis of certain antilithic remedies which were employed under different names. Another preparation, known as "Dutch drops," which is referred to in Paris's "Pharmacologia" (1822), and stated to consist of "oil of turpentine, tincture of guaiacum, spirits of nitric ether with oil of amber and cloves," was also largely used in the early part of last century. With the view of testing these preparations, I have for some years past given them a considerable trial in cases of gravel and stone, with results which sufficiently support the testimony to which reference has been made. The Dutch drops may be given in capsules containing 5 drops in doses of 10 to 30 drops a day. Though I am not prepared to state that their action is similar to that of common salt upon colloids, they appear to answer the purpose of aborting the stone-making process. In these ways, and possibly others, we may explain some of those spontaneous fractures of calculi which have been met with; urinary stones have before now been broken up and got rid of, and complete recovery followed without operation. In a short paper on this subject I reviewed several cases of this kind, where the evidence pointed to stones being expelled from the bladder after having undergone what was believed to be spontaneous disintegration or fracture. I referred to one case in particular where, after a long course of magnesian borocitrate, a patient I had seen who had been neither sounded nor operated upon in any way, passed sixty-four fragments of stone, weighing altogether two drachms. The fragments were composed of a hard uric acid core with a slight coating of phosphates. These fragments were all passed within an interval of about three months, and appear to have caused very little irritation. The angles of the fragments were for the most part rounded off as if they had been in the bladder some little time. The patient recovered, and attributed this result to the considerable amount of magnesian borocitrate he had consumed.

3. Rainey's discovery supplies an intelligible reason for practices which may appear empirical. Thus may be explained, apart from purely physical causes, the efficacy of such medicinal waters as Contrexéville, Evian, Wildungen, Harrogate, Llandrindod Wells, and other spas. In many instances the taking of large quantities of fluid at these resorts achieves the object by washing out stone and gravel from the urinary passages, much in the same way as drain-pipes are flushed when obstructed. In other instances something more than this is required, and may be effected by establishing and maintaining for some time an artificial condition of the urine.

I do not propose discussing the subject of diet in regard to the point under consideration. Relative to stone production diet should be judged not so much by any one constituent of a calculus, but rather by the whole concreting process. For instance, where the liability in an individual is marked, it is probable that the urinary colloids require quite as much watching as the crystals.

‡ Berlin. Klin. Woch., Nov. 3, 1879.

